# Neuralgia Pencils

**Published:** 1884-06

**Authors:** 


Neuralgia Pencils.—So-called neuralgia pencils are being sold by a number of German pharmacists. They are said to consist of a mixture of menthol, thymol, and eucalyptol, fused and cast in small conic pellets which are fitted in suitable handles. The forehead and temples being touched with the pencil, a slight impression of burning is at first produced, which gives way to a pleasant cool sensation. Several pharmacists claim priority in the invention.

